# Dental Age Assessment Based on Developmental Stages and Maturity Index of Third Molars for Medico-Legal Purposes

**DOI:** 10.3390/diagnostics14141559

**Published:** 2024-07-18

**Authors:** Petra Švábová, Michal Soták, Branislav Galis, Patrícia Kroupová, Lucia Bundová, Adriana Vojtušová, Soňa Masnicová, Radoslav Beňuš

**Affiliations:** 1 Department of Anthropology, Faculty of Natural Sciences, Comenius University, Ilkovičova 6, Mlynská dolina, 842 15 Bratislava, Slovakia; sotak11@uniba.sk (M.S.); kroupova4@uniba.sk (P.K.); adriana.vojtusova@gmail.com (A.V.); 2 Department of Oral and Maxillofacial Surgery, Faculty of Medicine, Comenius University and University Hospital Bratislava, Ružinovská 6, 826 06 Bratislava, Slovakia; brano.galis@gmail.com; 3 Institute of Forensic Medical Expertise, Expert Institute, 811 04 Bratislava, Slovakia; 4 Dental Surgery Clinic, University Hospital Bratislava, Pažítková 4, 821 01 Bratislava, Slovakia; 5 Department of Pre-Primary and Primary Education, Faculty of Education, Comenius University Bratislava, Šoltésovej 4, 811 08 Bratislava, Slovakia; bundova@fedu.uniba.sk; 6 Department of Criminalistics and Forensic Sciences, Academy of Police Forces, Sklabinská 1, 835 17 Bratislava, Slovakia; sona.masnicova@yahoo.com

**Keywords:** dental age, legal age, orthopantomograms, developmental stages of third molars, maturation index of third molars

## Abstract

As results may vary depending on the method of examination, this paper analyzes methods of age estimation based on the maturation index of third molars (I_3M_) and Kohler’s developmental stages in living individuals. A total of 1475 orthopantomograms were analyzed. The results showed that the development of third molars tended to be more advanced in males than in females. Regression equations that included the value of the developmental stage of the left third molar most accurately predicted chronologic age in males and females. Using the I_3M_ method, there were no statistically significant bilateral differences between the mandibular right and left third molars. Overall, 82.92% of males and only 72.82% of females were correctly classified with the cut-off value (0.08) for the left mandibular third molar index. In addition, 81.97% of individuals were correctly classified as minors and adults using the Kohler method, while only 78.62% of individuals were correctly classified as minors and adults using the I_3M_ method. Based on the misclassification of minors as adults, both methods should be used with caution and overestimation of age should be considered, especially for those close to 18 years of age.

## 1. Introduction

In the quest for unravelling the enigma of chronological age in living individuals, contemporary age estimation methods are cornerstones of scientific innovation, guiding us through the complexities of biological markers and cutting-edge technologies. Accurately predicting the chronological age of living individuals, i.e., the period of time that begins at birth and ends on a specific date, is an important goal in various fields, from forensics to clinical medicine and anthropological research. In contrast to chronological age, which progresses uniformly, biological age shows great variability among people due to genetic differences, lifestyle, environmental factors, and personal morbidities [1]. When estimating the age of living individuals, it is important to distinguish between chronological age, which is determined from the day of birth, and biological age, which represents the entire period of growth and development of an individual and is a measure of the development of its morphological and functional characteristics [1]. The term “dental age” refers to the developmental and eruption sequence of a person’s teeth [2]. The ability to estimate a person’s age is not only of great importance for legal proceedings and medical diagnoses, but also provides insight into the intricacies of population demographics. Reliable age estimation is essential for issuing legal identity documents in cases where birth records are not available, in immigration cases where verification of a minor asylum seeker is required, and in proceedings to assess the legal responsibility or status of a victim or suspect [3,4,5].

The first decades of this millennium have been characterized by significant progress in the development and refinement of age estimation methods. Dental age estimation is deemed a highly efficient and effective approach as it provides numerous advantages: Teeth tend to evolve in a well-documented, sequential manner and show relative consistency across populations, allowing for general categorization into stages [5]. Other favorable characteristics include their invulnerability towards environmental influences due to their high mineral content and lack of organic matter, malnutrition and disease, their adaptability to a wide age range, and a non-invasive mode of examination [6,7,8]. The third molars are the teeth utilized for age estimation owing to their late development, generally emerging between 17 and 25 years of age. This characteristic makes them particularly useful for age estimation in older adolescents and young adults [9,10].

There are numerous methods for estimating age, each of which works on a different basis. The method of Demirjian et al. [11] focuses on developmental criteria such as the deposition of dentin and the structure of the pulp chamber, the approaches of Haavikko and Moorrees use the stages of formation, and Nolla is concerned with the degree of mineralization. Gustafson performs the evaluation of several ground tooth sections, while Kohler and Cameriere use root development stages in their respective methods [12,13,14,15,16]. In contrast to the most widely utilized assessment by Demirjian et al. [11] which has been criticized for its overestimation of age, the methods of Cameriere and Kohler are generally less frequently criticized for this limitation [17]. The quantitative method of Cameriere et al. examines the apical roots of the third molars on the left side to determine the maturation index of the third molars. This index categorizes individuals as either over or under 18 years of age [18]. The approach of Kohler et al. is to correlate the developmental stages of the third molars with chronological age. The progression of third molar development is categorized into ten different stages, with each stage corresponding to a specific developmental phase numbered from one to ten [19].

Although a variety of approaches to estimating dental age have been formulated, the search for a method that accurately distinguishes between minors and adults and is applicable to all ethnicities persists. As the results may vary depending on the method used in the different studies, the present study aims to (i) perform a detailed analysis of Camerier and Kohler’s methods for estimating dental and chronological age in living individuals of Caucasian origin currently living in Central Europe, (ii) apply the regression equations of Mesotten et al. [20] to estimate chronological age and the age prediction equation calculated by the authors, and (iii) investigate possible differences in the development of the third molar with regard to the biological sex of the study sample and with regard to the location of the third molar in the dental arch, i.e., bilateral and collateral differences. The analyzed methods of estimating dental age based on OPGs of third molars could allow the classification of individuals as adults or minors by determining the percentage of probability of legal age, i.e., being 18 years and older, which is the age of criminal responsibility in Slovakia.

## 2. Materials and Methods

The present study was conducted retrospectively using data collected by dental practitioners from western and southwestern Slovakia. The orthopantomograms (OPGs) used in the present study were examined and analyzed by a single observer. All procedures complied with ethical standards. The third molars were numbered according to the two-digit FDI system, i.e., the right and left upper third molars as 18 and 28 and the right and left lower third molars as 38 and 48.

Participants’ data were stored confidentially with assigned identification numbers. A total of 1475 OPGs (661 females and 814 males, Table 1) were analyzed using the Digimizer and TomoCon Lite programs. Since we worked with a population of 1475, a confidence level of 95 (converted to a Z-score of 1.96), a standard deviation of 0.5, and a confidence interval (margin of error) of ±5%, our sample size should be 385, so the minimum number of samples required to meet the desired statistical constraints is 385 [21]. The analyzed sample consisted of individuals living in western and southwestern Slovakia. However, the ethnicity of the study group was not recorded, although it can be assumed that the participants were Caucasian. No information was obtained on the health status, genetic abnormalities, or social status of the individuals, as these were not the subject of investigation in our study. The general inclusion and exclusion criteria for both methods studied are listed below, while Section 2.1 and Section 2.2 contain further inclusion and exclusion criteria related to the method used.

The inclusion criteria were participants aged between 13 and 25 years at the time the OPGs were taken, and a sufficiently clear and legible orthopantomogram of the subjects, with both impacted and non-impacted third molars included in the study if their roots were radiographically distinguishable.

The exclusion criteria were OPGs of poor quality, subjects with an unknown date of birth, and subjects with obvious dental pathology on the panoramic radiograph in relation to the third molars.

### 2.1. Training and Validation Sample in the Method Based on Development Stages

In the training sample, only OPGs of individuals with third molars in both the maxilla and mandible were evaluated. The reduced sample that met the criteria for evaluating molars using the Kohler method consisted of 811 OPG images, of which 339 were female and 472 were male, with a mean age of 19.15 ± 2.35 (Table 1). The validation dataset consisted of 61 OPGs out of a total of 102 OPGs (Table 1), had to fulfill the same criteria as above, and were not included in the training sample.

**Figure 1:**
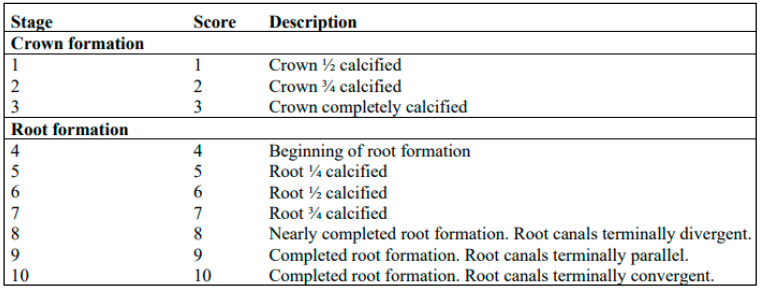
Explanation of crown and root development in the context of the progression of the developmental stages of the third molar, as presented by Kohler et al. [19] and Mesotten et al. [20].

In the Kohler method, the developmental stages of the third molars were correlated with the chronological age. Regression equations for predicting chronological age were then derived from the developmental stages of the third molars, which were evaluated using the Gleiser and Hunt [22] modification method described by Kohler et al. [19]. In this study, the developmental stages of all existing third molars were evaluated. The development of the third molar was categorized into ten developmental stages. Each stage corresponds to a specific developmental stage ranging from one to ten (Figure 1). In the case of a third molar with multiple roots, the least developed root was evaluated. The regression equations of Mesotten et al. [20] were used for further analysis.

### 2.2. Training and Validation Sample in Analyzed Third Molar Maturity Index (I_3M_) Method

Using the third molar maturity index (I_3M_) described by Cameriere et al. [18], only 968 OPGs met the criteria for the presence of both mandibular third molars, and the participants were between 13 and 25 years old. The selected sample that met the above criteria included 556 males and 412 females (Table 2). In addition, maxillary third molars were not examined due to frequent radiographic overlap with the maxillary tuberosity or difficulty in assessing the floor of the maxillary sinus [18,23]. The validation dataset had to fulfill the same criteria as above, was not included in the training sample, and comprised 72 OPGs (39 males and 33 females, Table 2).

**Figure 2:**
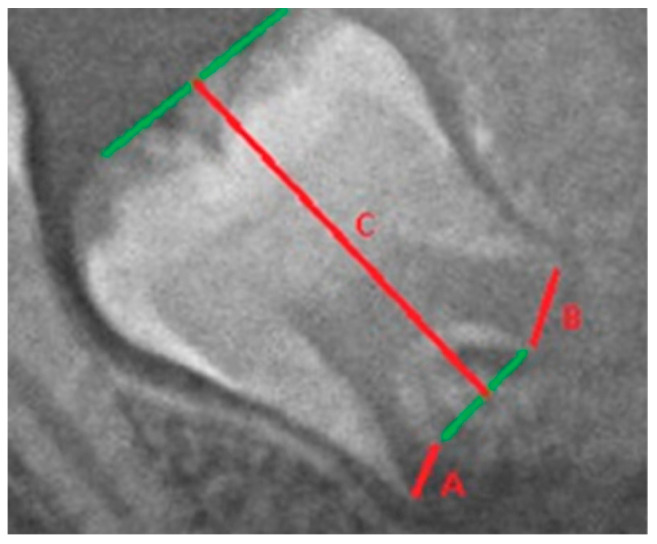
Measurements according to the method of Cameriere et al. [18]. Legend: A, B—root width, C—tooth height (Al-Qahtani et al. [27], modified).

The I_3M_ method is a metric method for assessing legal age. It uses the correlation between the actual age of an individual and standardized measurements that include the extent of open apices and the height of the third molar. The so-called maturation index of the third molars (I_3M_) was calculated with a cut-off value of 0.08. The method consists of evaluating the apical roots of the third molars on the left mandibular molars. If the root development of the third molar is complete, it means that the apical ends of the roots are completely closed, so the value of I_3M_ = 0.00. If the roots are open, I_3M_ is evaluated as (A + B)/C—the sum of the distances between the insides of two open apices (A + B) divided by the length of the tooth (C; Figure 2). The I_3M_ cut-off divides individuals into those older and younger than 18 years. Chronological age gradually decreases as the value of I_3M_ increases, i.e., if the value of I_3M_ < 0.08, the individual is categorized as 18 years old or older, and if I_3M_ ≥ 0.08, the individual is considered a minor [24,25,26].

### 2.3. Reliability and Reproducibility

To assess intrapersonal agreement, 50 randomly selected OPGs that simultaneously met the criteria for each method used were re-examined by the principal investigators two months later.

When using the I_3M_ method, measurement error was determined using quantitative variables based on the calculation of absolute technical error of measurement (TEM), relative technical error of measurement (rTEM), and reliability coefficient (R). The formula for calculating the relative value of TEM is as follows: rTEM = (TEM/VAV) × 100. The reliability coefficient (R) was calculated using the equation R = 1 − (TEM2/SD2). The R value ranges from 0 to 1 and the closer the R value is to 1, the more accurate the measurement is [28,29,30].

In the developmental stages, the intraoperator agreement was determined on the basis of qualitative variables using the kappa test. The resulting value of the kappa test can range from −1 to +1. The closer the result of the kappa test is to 1, the lower the measurement error between two measurements or between observers. The kappa value can also be negative; we interpret such a result as “no agreement”. Interpretation of the results of the kappa test is as follows [31]:

- a value ≤ 0 means no agreement;
- 0.01–0.20 => poor agreement;
- 0.21–0.40 => good agreement;
- 0.41–0.60 => moderate agreement;
- 0.61–0.80 => good agreement;
- 0.81–1.00 => very good/perfect agreement.

### 2.4. Statistical Analysis

Statistical analysis was performed using IBM SPSS Statistics 20, including descriptive analyses, normality distribution assessments, and visual inspections of histograms and q-q plots. A paired sample *t*-test was used for bilateral and collateral differences, while an independent sample *t*-test was used for differences between biological sexes. Linear regression equations, specific to males and females, used chronological age as the independent variable and third molar developmental stages as the dependent variable. The intrapersonal agreement of the developmental stages was assessed using Cohen’s kappa test and, in the case of the I_3M_ method, the technical error of measurement was calculated. The significance level was set at 5% so that all values below 0.05 were considered significant.

## 3. Results and Discussion

### 3.1. Analysis of the Developmental Stages of the Third Molars

The intrapersonal agreement was of very good agreement with a 95% CI. The kappa values ranged from 0.91 for the right maxillary third molar to 0.98 for the left mandibular third molar.

**Figure 3:**
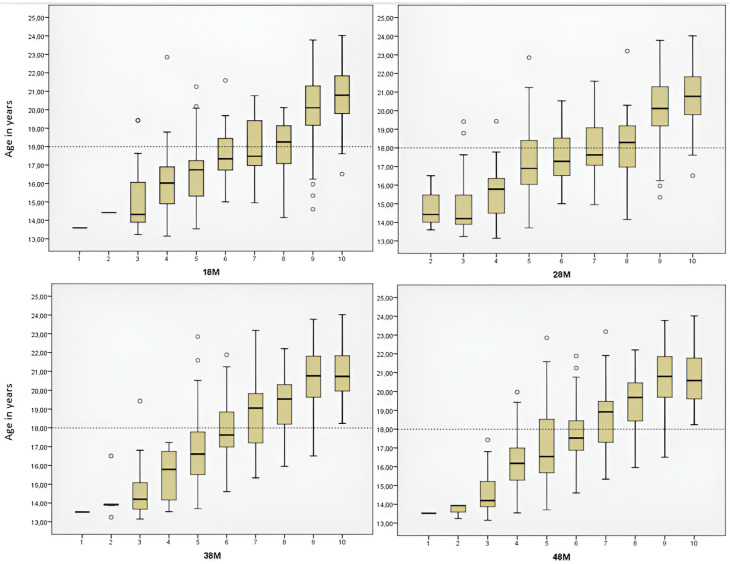
Age distribution for each third molar (18, 28, 38, 48) according to the developmental stages (from 1 to 10) in males.

**Figure 4:**
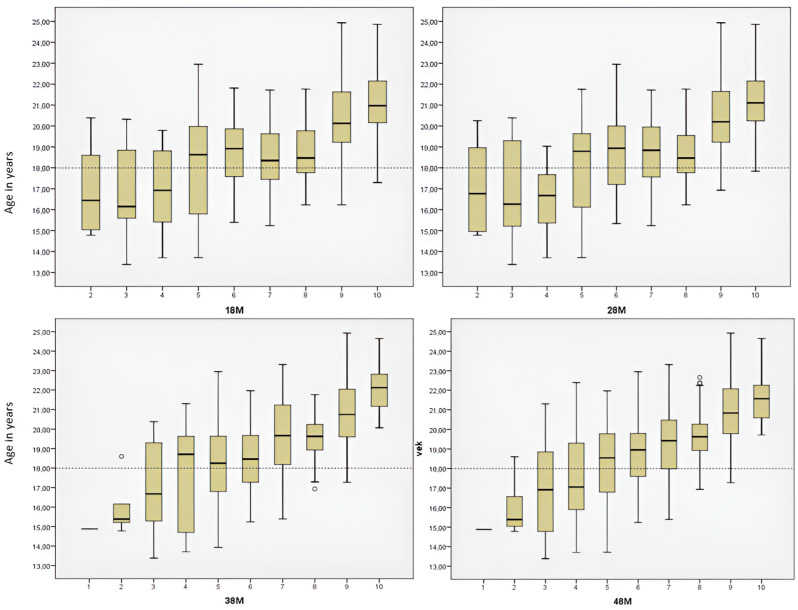
Age distribution for each third molar (18, 28, 38, 48) according to the developmental stages (from 1 to 10) in females.

Among males aged 18 years and older, a significant majority were in stage nine, which is particularly evident in Figure 3. Approximately 97.16% of males with a stage nine left third molar were 18 years or older, with similar results for the right mandibular third molar, where 97.08% of those in stage nine were in the same age group. In addition, all males with either the left or right mandibular third molar at stage ten were 18 years or older. Remarkably, differences were observed between mandibular and maxillary third molars at stage eight, as depicted in Figure 3. For maxillary third molars, the eighth stage occurs in both males aged 18 years and younger and males older than 18 years, whereas for mandibular third molars, it occurs only in males aged 18 years or older. The seventh stage is typically present when most individuals are 18 or older. Stage nine is most common in females aged 18 years and older (Figure 4). About 95.65% of females with a left third molar in stage nine were 18 years or older. Similarly, about 94.19% of females with the mandibular right third molar in stage nine were in the same age group. Females with either the right or left third molar at stage ten were all 18 years or older. It is noteworthy that there are differences between mandibular and maxillary third molars, especially in the seventh and eighth stages. In the study by Mesotten et al. [20], 17 males and 24 females under the age of 18 had at least one third molar in the tenth stage. In our study, one male and two females fell into this category, with an average age of 17.69 years, suggesting that third molar development may already be complete.

Statistically significant differences were found between the developmental stages of third molars in females and males (*p* < 0.05), meaning that third molar development tends to be more advanced in males than in females, which is consistent with the results of studies by Balla et al. [32], Scendoni et al. [33], Mesotten et al. [20], and Liversidge [34]. In contrast, Trakinienė et al. [35] argue that females experience an earlier maturation of the third molars compared to males. In addition, the present study found that the third molars in the maxilla develop significantly faster than the third molars in the mandible (*p* < 0.05). Additionally, there were no statistically significant disparities observed between the right and left mandibular or maxillary third molars (*p* > 0.05). Consequently, the developmental stages identified were used to estimate age using the equations proposed by Mesotten et al. [20] (Table 3). In males, the most accurate estimate was obtained by considering the developmental stage of the mandibular left third molar, which is reflected in the equation y = 13.0664 + 0.8006 × LL, giving an average age of 18.92 years, slightly underestimated by 0.08 years. For females, both equations gave comparable results. By substituting the developmental stage of either the maxillary or mandibular left third molar into the respective equations, we obtained an estimate for females with the equation y = 15.3523 + 0.5452 × LL, resulting in an average age of 19.15 years, with a slight underestimation of 0.23 years. These equations, which take into account the developmental stage of the left third molar, showed the best accuracy in age estimation for both sexes.

Due to the superior accuracy of the equations of Mesotten et al. [20], especially with respect to the developmental stage of the left mandibular third molar, linear regression equations were then calculated, tailored to this tooth (38M), separately for males and females. Subsequently, both the newly calculated linear regression equations and the equations of Mesotten et al. [20] were tested on a validation sample (Table 4). In the case of the Mesotten et al. [20] equations, the mean difference between the estimated and chronological age was −0.01 years for males and 0.11 years for females. In the equations developed by the authors of the present study, the difference between the mean values was 0.05 years for males and 0.35 years for females. The equation by Mesotten et al. [20] underestimated the age of males and overestimated the age of females. When using the authors’ equation, the age was slightly overestimated for both males and females.

Subsequently, the validation sample was divided into a group of minors (<18 years) and adults (>18 years) to determine the correct classification into adults or minors based on age calculated from above equations (Table 5). The results show that the classification of individuals was identical in both cases. The probability of being 18 years and older was 95.24% for females and 14 out of 14 males were correctly classified as adults. In the minor male group, the probability of being 18 years and older was 37.50% and in minor female group it was 40%. Although an error in which adult subjects are incorrectly assigned to the group of minors is not as serious as the assignment of minors to the adult group, some authors (Garamendi et al. [36]) have discussed the consequences of ethically and technically unacceptable errors. Technically unacceptable errors are associated with false negative results. In this way, subjects over the age of 18 are classified as minors and given preferential treatment by the judicial system. Ethically unacceptable errors are caused by false positives, which means that minors are treated as adults in terms of legal liability. As also stated in the study by Švábová et al. [37], ethically unacceptable errors should be eliminated and technically unacceptable errors should be reduced. The average age of boys who were misclassified as adults was 17.11 years, relatively close to the age limit of 18 years. Using the equation by the authors, the age in this group was overestimated by 1.08 years, which is lower than Mesotten et al.’s [20] equation, where the age was overestimated by 1.22 years. For females, the average age of misclassified girls was 15.01 years, which is not as close to the threshold for adulthood (18 years), but the average of the differences between estimated and chronological age is high in the group of minor girls. Using the authors’ equation, the age was overestimated by 3.53 years, which is less than using the equation of Mesotten et al. [20], where the age was overestimated by 3.62 years. Thus, the combination of the high average age of those misclassified as minors and the high average difference between the estimated and chronological ages may have caused the misclassification of minors into the adult group.

In the classification of females and males into the adult groups, i.e., 18 years of age, a borderline stage of development was identified. The estimated age of 18 years was when using developmental stage seven in the prediction equation for the males, but for the females it was developmental stage five. This suggests that teeth develop faster in males than in females, as also found in studies by Balla et al. [32], Scendoni et al. [33], Mesotten et al. [20] and Liversidge [34], but in contrast to the results of Trakinienė et al. [35]. The linear regression equations by both Mesotten et al. [20] and the authors classified the individuals equally into minor and adult groups. However, when looking at the mean values of the differences between the estimated and chronological ages for the entire group of males or females, regardless of the subdivision into minors and adults, the mean age of the differences is lower when using the Mesotten et al. [20] equation. When further dividing each group by sex into the minor and adult groups, the mean difference between the estimated and chronological ages using the authors’ equation is lower or closer to zero than the mean age differences using the Mesotten et al. [20] equations.

**Figure 5:**
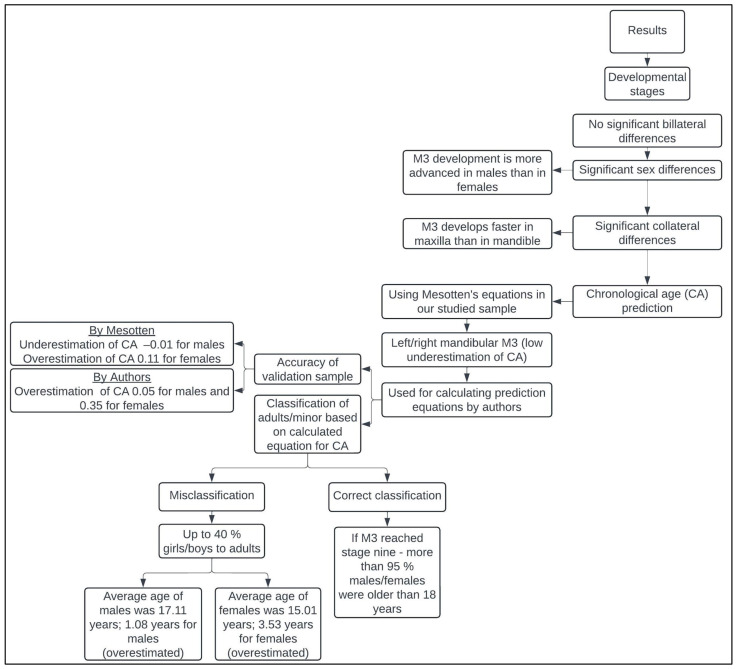
Flow chart showing the step-by-step progression of the results obtained by analyzing the developmental stages of the third molars.

The above results of the analysis of the developmental stages of the third molar are presented in a clear flow chart (Figure 5).

### 3.2. Analysis of I_3M_ Method

In the case of intrapersonal measurement error, the agreement between the two measurements is almost perfect. The lowest, but still highly significant, agreement was found for the dimension of the root width of the left mandibular third molar (R = 0.96). All the other dimensions showed a high degree of agreement (R = 0.99).

**Figure 6:**
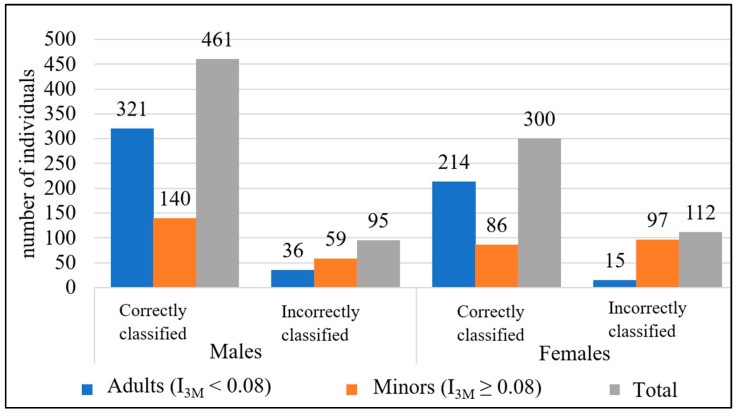
Age classification of individuals into minors and adults according to the index of maturity of the left third mandibular molar (I_3M_).

No statistically significant differences were found between the measurements of the right and left mandibular third molars (*p* > 0.05), except for the B width and the length of the third molar in males (Table 6) and in the case of the I_3M_ the bilateral differences were not statistically significant. The present results showed statistically significant differences between the sexes in the developmental progression of the third molars (*p* < 0.05) (Table 6). However, Cameriere et al. [18] established a cut-off value regardless of the sex of the individuals. The individuals were then stratified into two groups (minors and adults) based on the third molar index, with a cut-off value of 0.08 (Figure 6). With this cut-off value for the left mandibular third molar index, 82.92% of the males were correctly classified. Classification errors occurred mainly in minors, with up to 29.65% of minor boys being misclassified as adults. Among females, 72.82% were correctly classified based on the left mandibular third molar index threshold (I_3M_ = 0.08). However, of the minor girls, 53.01% were misclassified as adult females.

**Figure 7:**
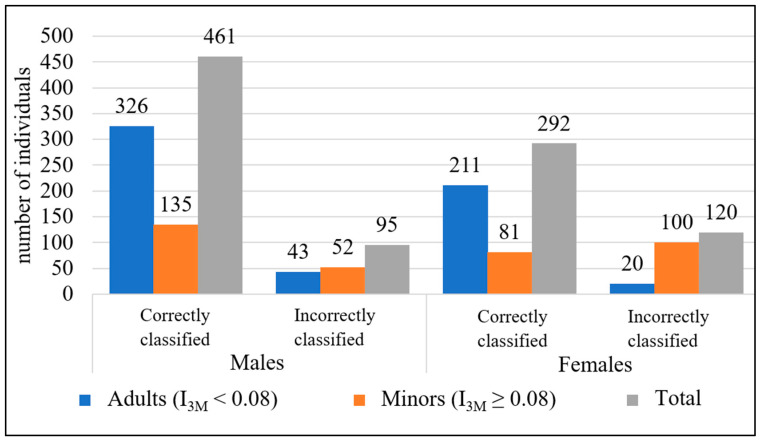
Age classification of individuals into minors and adults according to the index of maturity of the right third mandibular molar (I_3M_).

The individuals were then stratified according to the index of the third right mandibular molar (Figure 7). In males, 82.92% of the individuals were correctly classified, but 27.81% of the minor males were incorrectly classified as adults. For females, correct classification was achieved in 70.84% of cases. However, 55.23% of minor females were misclassified as adults.

The present results indicate that if the left molar is not available for assessment, the values of the right third molar can be used for age estimation, which is consistent with the results of Cameriere et al. [38] and De Luca et al. [39].

Table 7 depicts the results of the correct classification together with the population for which the threshold I_3M_ = 0.08 was tested. Cameriere et al. [18] determined an 83% correct classification rate for the third molar index (I_3M_) using individuals of the Caucasoid variety. Cavrić et al. [40] applied the I_3M_ to Black African population from Botswana and found no significant sex differences in the maturation of the left third molar. They achieved 94% correct classification for adolescent boys and 88% for adult males, with 88% for adult females and 96% for minor girls. Chu et al. [26] tested I_3M_ on a Chinese population and achieved 90.7% correct classification for males and 87.6% for females and found no differences in dental maturation between the sexes. Kalinowska et al. [41] tested I_3M_ on Polish individuals and obtained a correct classification of 87.6% for males and 85.3% for females. Moreover, a threshold of I_3M_ = 0.07 was also tested by Kalinowska et al. [41] and resulted in a correct classification of 86.5% for males and 84.4% for females. Cameriere et al. [42] classified females from 15 countries (Albania, Australia, China, Colombia, Dominican Republic, Egypt, France, Italy, India, Japan, Poland, Chile, Serbia, Turkey, South Africa) with I_3M_ = 0.08 and achieved classification rates of 91–98%. Spinas et al. [43] tested I_3M_ in an isolated Sardinian population and achieved an accuracy of 87% for males and 84% for females. Based on the above, it can be concluded that the I_3M_ method with a cut-off value of 0.08 is applicable in A various populations.

Based on the above results, the third molar index with a cut-off value of 0.08 is not very reliable, as a relatively large number of minors are classified in the adult group (especially among females). This could therefore mean that, with the Caucasian population at hand, it would make more sense to move the threshold down in a further analysis, as the correct classification of minors could then increase. As mentioned above and outlined by Garamedni et al. [36], these ethical errors caused by false positives should be eliminated so that minors are not treated as adults in terms of legal liability. As third molars are anatomically highly variable teeth whose roots do not lie on their longitudinal axis (the overall length of the tooth changes), they may affect the sensitivity of the method, which may have led to poor classification of individuals into age groups [51].

**Figure 8:**
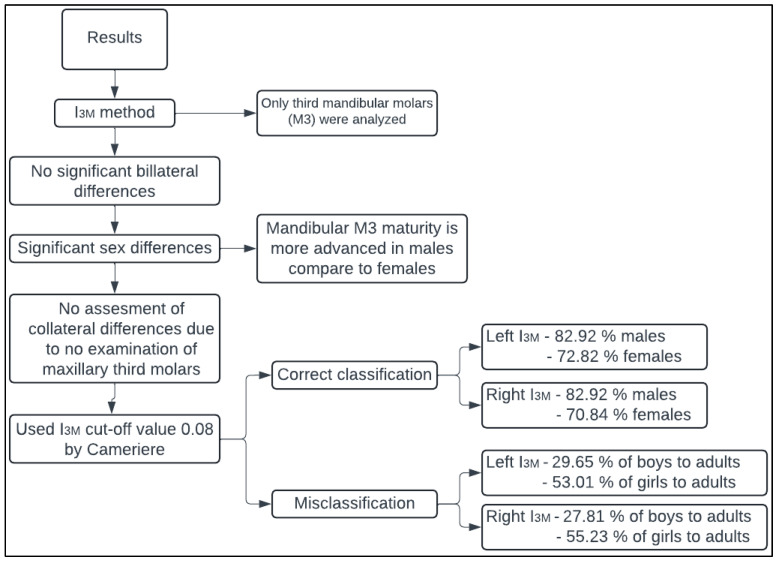
Flow chart showing the step-by-step progression of results obtained by analyzing the third molar maturity index method.

The step-by-step results of the above analysis of the third molar maturity index method are presented in a flow chart (Figure 8).

Although the study makes an important contribution to dental age assessment, there are some other limitations, including those mentioned above, that need to be emphasized, such as further analysis of a larger sample of OPGs of different age groups, including other regions of Slovakia or other countries and ethnic groups. Moreover, further research is needed, primarily concerned with comparing different methods to find one method or a combination of methods that most accurately correlate and predict chronological age and secondly, most importantly, eliminate the misclassification of minors as adults.

## 4. Conclusions

In summary, the results obtained show that the development of third molars tends to be completed earlier in males than in females. Furthermore, it is possible to use the maturation index of the third molars or the developmental stages of either the left or right third molars, as there are no significant bilateral differences. However, the development/mineralization of the mandibular third molars differed from that of the maxillary third molars, which means that the collateral differences were significant. Based on Kohler’s developmental stages, 81.97% of individuals were correctly classified as minors or adults. The authors’ age prediction equations yielded lower mean differences in classification as adults or minors compared to the prediction equations used by Mesotten [20], although the inclusion of minors in the adult group was considered ethically unacceptable. The complexity of the method, based on the maturation index of the third molar, resulted in only 78.62% correct classification of individuals as adults or minors. Given the notable misclassification of minors as adults, both methods should be used with caution and overestimation of age should be considered, especially for individuals who are close to 18 years of age.

## Figures and Tables

**Table 1:** Baseline characteristics of the training and validation for developmental stages method.

	Training Sample			Validation Sample		
	Males	Females	Total	Males	Females	Total
total number of analyzed OPGs	814	661	1475	49	53	102
reduced sample *	472	339	811	30	31	61
mean age in reduced sample *	18.99	19.38	19.15	18.84	19.09	18.97
standard deviation in reduced sample *	2.44	2.20	2.35	3.05	3.85	3.45
minimal age in reduced sample *	13.15	13.39	13.15	13.48	13.14	13.14
maximal age in reduced sample *	24.03	24.93	24.93	24.98	24.17	24.98

Legend: * number of OPGs that met the criteria for further analysis according to the Kohler and Mesotten method.

**Table 2:** Baseline characteristics of the training and validation for I_3M_ method.

	Training Sample			Validation Sample		
	Males	Females	Total	Males	Females	Total
total number of analyzed OPGs	814	661	1475	49	53	102
reduced sample *	556	412	968	39	33	72
mean age in reduced sample*	19.24	18.66	19.16	19.26	19.25	19.26
standard deviation in reduced sample *	2.82	2.90	2.32	3.00	3.78	3.35
minimal age in reduced sample *	13.50	13.15	13.15	13.48	13.14	13.14
maximal age in reduced sample *	24.98	24.17	24.93	24.98	24.17	24.98

Legend: * number of OPGs that met the criteria for further analysis according to I_3M_ method.

**Table 3:** Age estimation based on the developmental stages of each third molar in the studied sample according to the equations of Mesotten et al. [20].

Males with Mean Age 18.99 Years, SD 2.44		
Equations	Estimated Age in Years	MD
y = 13.4694 + 0.7524 × UL	19.32	0.33
y = 15.9933 + 0.4494 × LR	19.27	0.28
y = 13.0664 + 0.8006 × LL	18.92	−0.07
y = 13.0189 + 0.4613 × UL + 0.3785 × LR	19.37	0.38
y = 10.2000 + 0.5122 × UL + 0.5276 × LL	018.04	−0.95
**Females with Mean Age 19.38 Years, SD 2.20**		
**Equations**	**Estimated Age in Years**	**MD**
y = 15.5810 + 0.6057 × UR	20.11	0.72
y = 13.9157 + 0.6986 × UL	19.15	−0.23
y = 15.2038 + 0.5507 × LR	19.01	−0.38
y = 15.3523 + 0.5452 × LL	19.15	−0.23
y = 14.1709 + 0.6688 × LR	18.79	−0.59
y = 13.6206 + 0.1933 × UR + 0.5080 × LR	18.58	−0.80

Legend: Mean—mean age, MD—mean differences, LL—left mandibular third molar, UL—left maxillary third molar, LR—right mandibular third molar, UR—right maxillary third molar.

**Table 4:** Age estimation based on the developmental stages of the mandibular left third molar in the studied sample according to the equations of Mesotten et al. [20] and the authors (2024).

Equations by Mesotten et al. [20]		
	Estimated Age in Years	MD
Males (38M)		
AGE = 13.0664 + 0.8006 × LL	18.83	−0.01
Females (38M)		
AGE = 15.3523 + 0.5452 × LL	19.20	0.11
**Equations by Authors (2024)**		
Males (38M)		
AGE = 12.119 + 0.941 × 38M	18.89	0.05
Females (38M)		
AGE = 14.705 + 0.670 × 38M	19.44	0.35

Legend: Mean—mean age, MD—mean differences, LL, 38M—left mandibular third molar.

**Table 5:** The classification of individuals into minors and adults based on age prediction according to the equations of Mesotten et al. [20] and the authors (2024).

Equations by Mesotten et al. [20]										
	Males (N = 30)					Females (N = 31)				
	R	%	W	%	Total	R	%	W	%	Total
Minors	10	62.5	6	37.5	16	6	60	4	40	10
Adults	14	100	0	0	14	20	95.24	1	4.76	21
**Equations by Authors (2024)**										
	**Males (N = 30)**					**Females (N = 31)**				
	**R**	**%**	**W**	**%**	**Total**	**No. of R**	**%**	**W**	**%**	**Total**
Minors	10	62.5	6	37.5	16	6	60	4	40	10
Adults	14	100	0	0	14	20	95.24	1	4.76	21

Legend: R—number of correctly classified individuals, W—number of incorrectly classified individuals.

**Table 6:** Bilateral and intersex differences in I_3M_ values and measurements of third molars.

Third Molar and I_3M_	MALES		BD	FEMALES		BD	ID
	Mean	SD	*p*-Values	Mean	SD	*p*-Values	*p*-Values
A 38	13.30	25.64	0.448	15.39	24.04	0.609	<0.001
A 48	14.13	26.99		15.08	22.88		<0.001
B 38	5.15	7.08	0.000	5.94	6.69	0.176	0.005
B 48	4.41	5.97		5.59	6.11		<0.001
C 38	94.71	87.90	0.000	114.32	82.82	0.076	0.002
C 48	92.27	85.70		118.24	79.41		<0.001
I_3M_ 38	0.14	0.28	0.269	0.17	0.26	0.546	<0.001
I_3M_ 48	0.16	0.33		0.16	0.24		<0.001

Legend: I_3M_—third molar maturity index, A, B—root width of third molar, C—third molar height, 38—left mandibular third molar, 48—right mandibular third molar, BD—bilateral differences = differences between right and left mandibular molars in analyzed parameters, ID—differences between sexes in analyzed parameters, SD—standard deviation.

**Table 7:** Classification rates of the third molar maturity index (I_3M_) in different populations.

Study	Country/Population	Correct Classification Rate	
		Males	Females
Cameriere et al. [18]	Caucasoids	83.00%—regardless sex	
Gulsahi et al. [44]	Turkey	97.60%	92.70%
Cavrić et al. [40]	Black (Botswana)	91.00%	92.00%
Chu et al. [26]	China	90.70%	87.60%
Kalinowska et al. [41]	Poland	87.60%	85.30%
Spinas et al. [43]	Sardinia	87.00%	84.00%
Ribier et al. [45]	France	90.60%	80.70%
Authors (2024)	Slovakia (Caucasoids)	82.92%	72.82%
Doğru et al. [46]	Netherlands	88.90%	83.30%
De Moraes Correia et al. [47]	Brazil	84.80%	74.80%
Sharma et al. [48]	India	89.10%	87.50%
Cameriere et al. [38]	Albania	92.50%	87.50%
Al-Qahtani et al. [27]	Saudi Arabia	75.60%	72.40%
Galić et al. [49]	Croatia	91.50%	88.80%
Zelic et al. [50]	Serbia	95.00%	91.00%
Da Nóbrega et al. [51]	Brazil	84.30%	76.60%
Tafrount et al. [52]	France	91.60%	89.70%
Kelmendi et al. [53]	Kosovo	96.80%	90.90%
Antunović et al. [54]	B Montenegro	93.00%	89.00%

## Data Availability

The authors confirm that the data supporting the findings of this study are available within the article.
